# 2-Hydroxyglutarate Production, but Not Dominant Negative Function, Is Conferred by Glioma-Derived NADP^+^-Dependent Isocitrate Dehydrogenase Mutations

**DOI:** 10.1371/journal.pone.0016812

**Published:** 2011-02-04

**Authors:** Genglin Jin, Zachary J. Reitman, Ivan Spasojevic, Ines Batinic-Haberle, Jian Yang, Oleg Schmidt-Kittler, Darell D. Bigner, Hai Yan

**Affiliations:** 1 The Preston Robert Tisch Brain Tumor Center at Duke, Pediatric Brain Tumor Foundation Institute, and Department of Pathology, Duke University Medical Center, Durham, North Carolina, United States of America; 2 Clinical Pharmacology Laboratory, Department of Medicine, Duke Comprehensive Cancer Center, Duke University Medical Center, Durham, North Carolina, United States of America; 3 Department of Radiation Oncology, Duke University Medical Center, Durham, North Carolina, United States of America; 4 The Sidney Kimmel Comprehensive Cancer Center, Johns Hopkins University School of Medicine, Baltimore, Maryland, United States of America; The University of Chicago, United States of America

## Abstract

**Background:**

Gliomas frequently contain mutations in the cytoplasmic NADP^+^-dependent isocitrate dehydrogenase (IDH1) or the mitochondrial NADP^+^-dependent isocitrate dehydrogenase (IDH2). Several different amino acid substitutions recur at either IDH1 R132 or IDH2 R172 in glioma patients. Genetic evidence indicates that these mutations share a common gain of function, but it is unclear whether the shared function is dominant negative activity, neomorphic production of (R)-2-hydroxyglutarate (2HG), or both.

**Methodology/Principal Findings:**

We show by coprecipitation that five cancer-derived IDH1 R132 mutants bind IDH1-WT but that three cancer-derived IDH2 R172 mutants exert minimal binding to IDH2-WT. None of the mutants dominant-negatively lower isocitrate dehydrogenase activity at physiological (40 µM) isocitrate concentrations in mammalian cell lysates. In contrast to this, all of these mutants confer 10- to 100-fold higher 2HG production to cells, and glioma tissues containing IDH1 R132 or IDH2 R172 mutations contain high levels of 2HG compared to glioma tissues without IDH mutations (54.4 vs. 0.1 mg 2HG/g protein).

**Conclusions:**

Binding to, or dominant inhibition of, WT IDH1 or IDH2 is not a shared feature of the IDH1 and IDH2 mutations, and thus is not likely to be important in cancer. The fact that the gain of the enzymatic activity to produce 2HG is a shared feature of the IDH1 and IDH2 mutations suggests that this is an important function for these mutants in driving cancer pathogenesis.

## Introduction

Heterozygous point mutations in IDH1 and IDH2 occur in a significant portion of human cancers. Affected cancer types include gliomas of intermediate malignant grade (73–94%) [1], [2] and acute myeloid leukemias (AMLs, 16–22%) [3], [4], [5], [6], and cases of IDH1 mutations have been reported in prostate cancer [7], acute lymphoblastic leukemia, B type [7], colorectal cancer [8], and melanoma [9]. The IDH1 mutations observed in cancer tissue are specific for R132, a residue in the enzyme active site. R132H is the most common IDH1 substitution in gliomas, followed by R132C, R132S, R132L, and R132G. IDH2 is homologous to IDH1, and IDH2 mutations in glioma are specific for R172, the residue that is analogous to IDH1 R132. R172K, R172M, and R172G are the IDH2 substitions observed in gliomas. The IDH2 R172 mutations are rarer than the IDH1 R132 mutations in gliomas, and mutations in either gene are mutually exclusive in cancer (for reviews, see [10], [11]). The frequent observation of heterozygous hotspot mutations in IDH1 and IDH2 suggests that they are proto-oncogenes that are activated by these mutations in cancer. In line with this, two different molecular gains-of-function have been demonstrated for several of the mutated forms of IDH1 and IDH2.

The first proposed function for IDH mutants is dominant negative inhibition of WT IDH enzymes. IDH1 R132 and IDH2 R172 mutations inactivate the normal NADP^+^-IDH activity of IDH1 and IDH2 to convert isocitrate to α-ketoglutarate [2], [12]. Furthermore, IDH1-R132H can bind to IDH1-WT, and the resulting WT:mutant heterodimer has markedly lowered isocitrate dehydrogenase activity at physiological isocitrate concentrations (<80 µM) compared to WT:WT homodimers in vitro [13].

Since the IDH mutations observed in cancer are heterozygous, it has been speculated that IDH1 and IDH2 mutants bind the remaining IDH1-WT or IDH2-WT molecules in cells and exert a dominant negative function. However, it is unclear whether binding to IDH1-WT or IDH2-WT is shared by all of the glioma-derived IDH mutants, or whether IDH mutants actually bind a significant portion of IDH1-WT or IDH2-WT molecules to exert this dominant negative function in cells. In one study, tumor tissue from glioblastomas with IDH1 R132 mutations had 38% lower NADP^+^-IDH activity on average than tissue from glioblastomas without IDH mutations [14]. However, it is unclear whether this lowered activity reflects the loss of activity from the mutated IDH1 allele, or if it also reflects dominant negative lowering of the activity from the remaining WT IDH1 allele or the WT IDH2. The second proposed function for IDH mutants is that they gain the neomorphic activity to reduce α-ketoglutarate to 2HG [4], [6], [15]. However, this has not been demonstrated for all of the recurrent IDH mutants, nor has it been determined whether IDH2-mutated gliomas contain elevated 2HG levels.

The relative importance of either dominant negative activity or 2HG-producing catalytic activity for the IDH1 and IDH2 mutants in cancer is unclear, and the mechanism by which either of these gained functions may contribute to cancer pathogenesis remains unknown. Examining the relevance of these two molecular functions has the potential to identify therapeutic targets for cancer treatment, and to inform future studies on the mechanism of cancer pathogenesis for IDH-mutated cancer cells. To resolve this issue, we focus on identifying shared functions for the IDH mutations in cancer, and ruling out functions that are not shared, for a panel of eight glioma-derived IDH mutants. We reasoned that functions of the IDH mutants that are important in cancer will be shared between all eight mutants, and that functions that are not required for cancer pathogenesis may not be shared. We show that binding to, and dominant negative inhibition of, IDH1-WT or IDH2-WT is not a shared feature of the glioma-derived IDH mutants, but that 2HG production and accumulation in tumors is a common feature of these mutants.

## Results

### IDH1 R132 mutants bind IDH1-WT, but IDH2 R172 mutants poorly bind IDH2-WT

**Figure 1:**
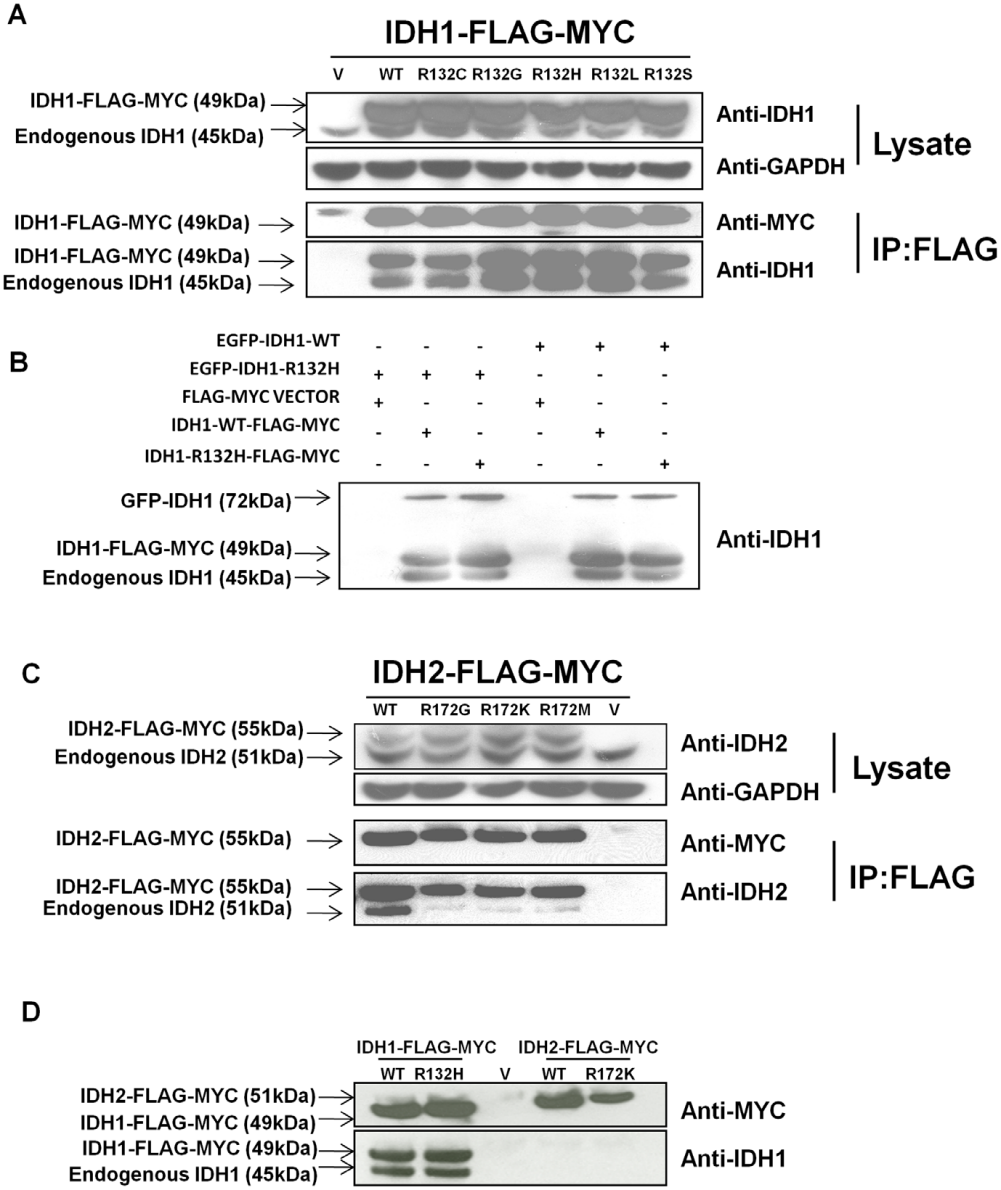
IDH1 R132 mutants bind IDH1-WT, but IDH2 R172 mutants poorly bind IDH2-WT. (a) Whole cell lysates of HOG cells overexpressing FLAG-MYC-tagged IDH1 proteins contain comparable amounts of endogenous IDH1. FLAG-IP of these lysates coprecipitates endogenous IDH1-WT (lower anti-IDH1 band) along with the FLAG-MYC-tagged IDH1 proteins (anti-MYC band and upper anti-IDH1 band). (b) HOG cells were transfected with FLAG-MYC-tagged IDH1-WT, IDH1-R132H, or a vector control (V) in combination with EGFP-tagged IDH1-WT or IDH1-R132H. As expected, FLAG-IPs of these cells pull down FLAG-MYC-tagged IDH1 (middle band). Endogenous IDH1 (lower band) coprecipitates with FLAG-MYC-tagged IDH1. EGFP-tagged IDH1 (upper band) also coprecipitates with FLAG-MYC-tagged IDH1. (c) Whole cell lysates of HOG cells overexpressing FLAG-MYC-tagged IDH2 contain comparable amounts of endogenous IDH2. FLAG-IP of these lysates coprecipitates endogenous IDH2 (anti-IDH2, lower band) along with the FLAG-MYC-tagged IDH2 (anti-IDH2, upper band and anti-MYC band). However, IDH2 R172 mutants pull down endogenous IDH2-WT poorly compared to IDH2-WT. (d) FLAG-MYC-tagged IDH1-WT, IDH1-R132H, IDH2-WT, and IDH2-R172K were transfected into HOG cells. FLAG-IP of lysates pulled down FLAG-MYC-tagged IDH1-WT, IDH1-R132H, IDH2-WT, and IDH2-R172K (anti-MYC bands). FLAG-MYC-tagged IDH1-WT and IDH1-R132H also stained with anti-IDH1 (upper anti-IDH1 band). Endogenous IDH1-WT coprecipitated with FLAG-MYC-tagged IDH1-WT and IDH1-R132H, but not with FLAG-MYC-tagged IDH2-WT and IDH2-R172K (lower anti-IDH1 band).

IDH1 and IDH2 function as homodimers, and IDH1 R132H can bind to and dimerize with WT IDH1 *in vitro* [13]. However, it is unclear whether other IDH1 R132 mutants can bind IDH1. To determine this, we overexpressed IDH1-WT or one of five glioma-derived IDH1 R132 mutants with C-terminal MYC and FLAG tags in human oligodendroglioma (HOG) cells, a human glioma cell line that does not contain IDH mutations [2]. We then determined whether endogenous IDH1-WT coprecipitated with the overexpressed forms of IDH1 by performing anti-FLAG immunoprecipitation. Transgenic IDH1-WT and IDH1 R132 mutants were similarly able to bind and pull down endogenous IDH1-WT (Fig. 1a). We next tested whether IDH1-R132H, the most common IDH mutant in gliomas [2], could bind to other IDH1-R132H molecules in cells. To do so, we first transfected glioma cells with combinations of IDH1-WT and IDH1-R132H with MYC-FLAG or EGFP tags. Then, we assayed the ability for MYC-FLAG-tagged IDH1-WT or IDH1-R132H to pull down EGFP-tagged IDH1-WT or IDH1-R132H. The formation of IDH1 WT:R132H, WT:WT, and R132H:R132H was about equal in this system (Fig. 1b).

Since IDH1 R132 mutants can bind IDH1-WT, we hypothesized that the analogous IDH2 R172 mutants could bind IDH2-WT. However, IDH2 R172 mutants were only weakly able to pull down endogenous IDH2 compared to IDH2-WT in these cells (Fig. 1c). Since binding to IDH1-WT may be an important function for IDH1 R132 mutants in cancer [13], we hypothesized that IDH2 R172 mutants may localize outside the mitochondria and also bind IDH1-WT. However, neither IDH2-WT nor IDH2-R172K was able to bind and pull down endogenous IDH1, indicating that IDH1 and IDH2 cannot bind to each other (Fig. 1d).

### IDH mutants do not dominantly lower cellular IDH activity

Purified IDH1-R132H:WT heterodimers have a lowered isocitrate dehydrogenase reaction rate compared to IDH1-WT:WT homodimers at low isocitrate concentration [13]. Based on this, IDH1 and IDH2 mutants have been proposed to inhibit the activity of the wild-type IDH1 or IDH2 allele in cancer cells in a dominant-negative fashion. However, it is unknown whether IDH1-R132H or other IDH1 and IDH2 mutants can lower the activity of wild-type IDH1 or IDH2 in cells. Previous studies of the effect of IDH1 and IDH2 mutant overexpression on endogenous isocitrate dehydrogenase activity in cell lysates have been carried out at relatively high substrate concentrations (400 µM or 1.3 mM) [2], [12]. At these high concentrations, mutant:WT heterodimers have a similar reaction rate to WT:WT homodimers [13]. Thus, in these studies, any dominant negative activity exerted by mutant enzymes on the wild-type endogenous proteins would be masked.

**Figure 2:**
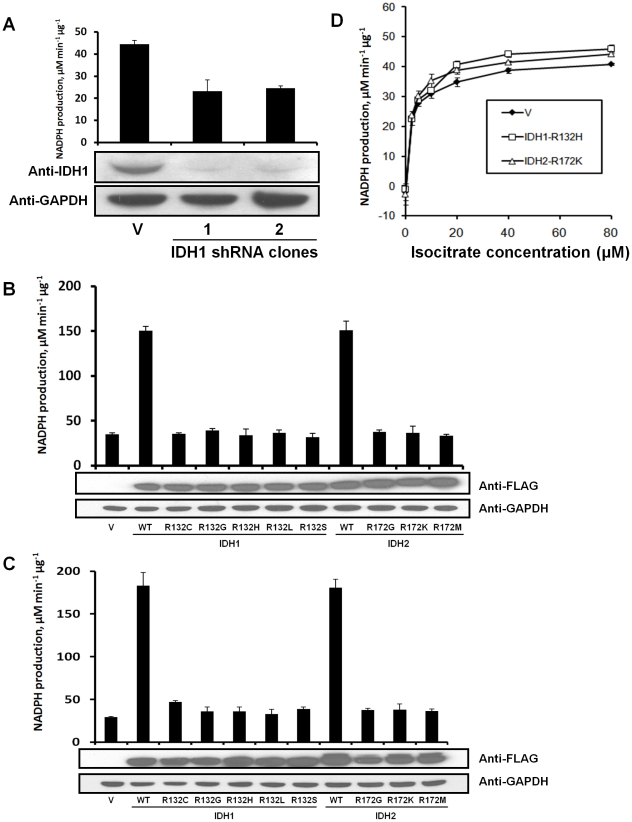
Overexpression of IDH1 R132 and IDH2 R172 mutants does not reduce cellular NADP^+^-IDH activity. Total NADP^+^-IDH reaction rate, which consists of the combined rates of IDH1 and IDH2, was measured in lysates by measuring the rate of conversion of NADP^+^ to NADPH in the presence of isocitrate. (a) Lysates of stable, clonal HOG cell lines containing an shRNA sequence targeted against IDH1 have lowered NADP^+^-IDH activity compared to a sister cell line expressing scrambled RNA from the same vector (V). (b) HOG cells were transfected with a vector control, IDH1-WT, an IDH1 R132 mutant, IDH2-WT, or an IDH2 R172 mutant, lysates were collected after 48 h, and total cellular NADP^+^-IDH reaction rate at 40 µM isocitrate was assayed. (c) The experiment in *B* was repeated with 293T cells. (d) 293T lysates transfected with vector control, IDH1-R132H, or IDH2-R172K from *C* were assayed for NADP^+^-IDH reaction rate at 0 to 80 µM isocitrate.

Both IDH1 and IDH2 have NADP^+^-dependent isocitrate dehydrogenase (NADP^+^-IDH) activity, and no other mammalian enzymes have this activity. Because of this, assays of NADP^+^-IDH activity on whole cell lysates reflect the combined activity of IDH1 and IDH2. To provide information on the proportion of NADP^+^-IDH activity contributed by IDH1 and by IDH2 in cells, we stably transfected HOG cells with IDH1 shRNA or scrambled shRNA. IDH1 expression was reduced by 90 percent in the IDH1 shRNA cells, and the NADP^+^-IDH activity was reduced by nearly half, indicating that IDH1 accounts for nearly half of cellular NADP^+^-IDH activity (Fig. 2a). The remaining activity in IDH1 knockdown cells may be supplied by the residual IDH1 enzyme and by IDH2.

To determine whether IDH1 and IDH2 mutants can dominantly inhibit the activity of endogenous IDH1 and IDH2 enzymes, we assayed NADP^+^-IDH activity of lysates of HOG cells overexpressing IDH1-WT, IDH2-WT, or cancer-derived IDH1 R132 and IDH2 R172 mutants in the presence of 40 µM isocitrate. Importantly, FLAG-MYC-tagged IDH1-WT and IDH2-WT have previously been shown to have their native enzyme activity in this setting [2], indicating that dimerization and enzymatic activity are not affected by this epitope tag. 40 µM isocitrate closely mimics the concentration of this metabolite in cells [16]. Additionally, purified IDH1 R132H:WT heterodimers have a significantly lower reaction rate than WT:WT homodimers at this substrate concentration [13]. We therefore reasoned that any inhibition of endogenous wild-type IDH1 or IDH2 by transgenic IDH1 or IDH2 mutants would be apparent at this substrate concentration. As expected, overexpession of IDH1-WT or IDH2-WT greatly increased the total NADP^+^-IDH reaction rate at 40 µM isocitrate (Fig. 2b). However, transgenic IDH1 R132 and IDH2 R172 mutants had little effect on this activity (Fig. 2b), showing that they do not dominant-negatively inhibit IDH1-WT or IDH2-WT in this system. To validate this finding and expand it to non-cancer cells, we repeated this experiment in 293T cells and found similar results (Fig. 2c).

To test whether overexpression of cancer-derived mutants of IDH1 or IDH2 could reduce total cellular NADP^+^-IDH activity at other concentrations of isocitrate at which mutant:WT heterodimers have lowered activity, we also assayed the total cellular NADP^+^-IDH activity of the 293T cells expressing IDH1-R132H or IDH2-R172K described above over a range of 0 to 80 µM isocitrate. These mutants did not lower the overall NADP^+^-IDH activity compared to vector alone at any of the substrate concentrations we assayed (Fig. 2*d*). These data indicate that homologous expression of IDH mutants does not interfere with normal, endogenous IDH1-WT or IDH2-WT. However, it is possible that homologously expressed IDH mutants could have limited interaction with endogenous IDH in cells. To address this possibility, we determined whether an IDH1 R132 mutant could inhibit IDH1-WT when both are expressed homologously. To do so, we co-expressed IDH1-R132H and IDH1-WT in HOG cells, and found that IDH1-R132H expression did not lower cellular NADP^+^-IDH activity of cells expressing IDH1-WT more than vector alone (Fig. S1).

### IDH mutants produce 2HG, which accumulates in IDH-mutated gliomas

**Figure 3:**
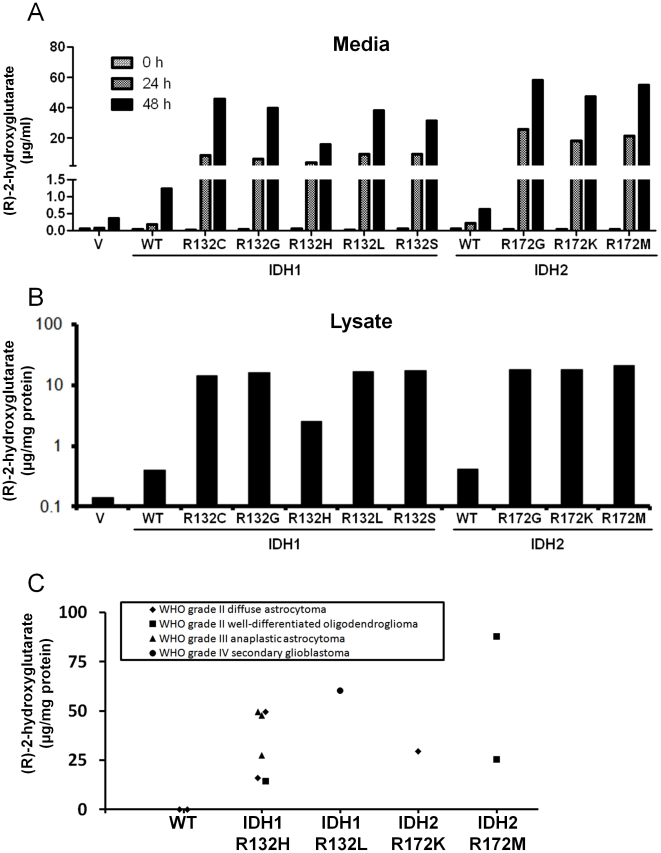
IDH1 R132 and IDH2 R172 mutants produce 2HG. (a) HOG cells were transfected with an empty vector (V), IDH1-WT, the indicated IDH1 R132 mutant, IDH2-WT, or the indicated IDH2 R172 mutant. The media was changed after 24 h. 2HG was analyzed in samples of media taken at 0, 24, and 48 h after the media was changed. (b) 2HG was measured in lysates of HOG cells expressing the indicated IDH variant 48 hours after transfection. (c) 2HG levels in human gliomas with either no IDH mutation (WT) or the indicated mutation.

Overexpression of IDH1-R132H, as well as IDH2-R172K, in mammalian cells leads to accumulation of 2HG in culture media [6], [15]. To confirm this finding and expand it to the other IDH mutants found in gliomas, we measured 2HG levels in media incubated with HOG cells overexpressing IDH1-WT, IDH2-WT, or one of the recurrent cancer-derived mutants. To measure 2HG, we adapted a protocol to use derivation with diacetyl-L-tartaric anhydride to separate (R)-2-hydroxyglutarate, which is produced by IDH mutants, from its enantiomer, (S)-2-hydroxyglutarate, on a liquid chromatography (LC) column before performing tandem mass spectrometry (MS/MS) [17]. (R)-2-hydroxyglutarate accumulated over time in culture media for all mutants assayed (Fig. 3*a*). To confirm this, we measured 2HG levels in media incubated with 293T cells overexpressing the same IDH proteins and found similar results (data not shown). We also determined the levels of 2HG in lysates of HOG cells expressing IDH1 R132 and IDH2 R172 mutants and found that 2HG was 10- to 100-fold elevated in these lysates compared to vector alone (Fig. 3*b*)

Gliomas containing IDH1 R132 mutations have marked elevations of 2HG [15]. Also, AMLs containing IDH1 R132 or IDH2 R172 mutations have marked elevations of 2HG [4], . We therefore reasoned that gliomas bearing IDH2 R172 mutations would also have elevated 2HG. To test this, we analyzed 2HG in 12 glioma samples, including samples containing IDH1 R132H, IDH1 R132L, IDH2 R172K, IDH2 R172M or no IDH mutations. We used a deuterated internal standard, [3,3,4,4-^2^H_4_]-(R/S)-2-hydroxyglutarate (2HG-d4), to reliably quantify 2HG in glioma tissue samples as described previously [17]. We found that all tumors containing IDH mutations had markedly elevated (R)-2-hydroxyglutarate compared to tumors without IDH mutations (54.4 vs. 0.1 mg 2HG/g protein, p<0.001, Fig. 3c). This demonstrates for the first time to our knowledge that gliomas with IDH2 R172 mutations can have elevated 2HG, that any cancer with IDH1 R132L or IDH2 R172M have elevated 2HG, and that 2HG in cancer tissues is specific for (R)-2-hydroxyglutarate.

## Discussion

IDH1 and IDH2 mutations arise early in cancer pathogenesis and are specific for hotspot codons, suggesting that these mutations have a shared oncogenic function that drives cancer pathogenesis. We show that IDH mutants bind their corresponding WT partners either approximately the same as (IDH1) or more poorly than (IDH2) the WT version of the protein (Fig. 1a,c). Since the property of binding to a WT partner is not shared among the IDH mutants, it is unlikely that binding to a WT partner is an important feature of the IDH mutations in driving cancer pathogenesis.

Also, our data shows that IDH mutants do not exert a dominant negative function in a model for IDH-mutated cancer cells. We have shown that IDH1 WT:WT, WT:mutant, and mutant:mutant dimers form in cells that express both mutant and WT forms of IDH1 (Fig. 1b). Based on this, cancer cells that have heterozygous IDH1 or IDH2 mutations would be expected to contain a mixture of WT:WT, WT:mutant, and mutant:mutant IDH1 or IDH2 dimers. A previous study showed that the IDH1 WT:R132H heterodimer has a lowered NADP^+^-IDH activity at low concentrations of isocitrate [13]. Because the IDH1 R132 mutant must bind IDH1-WT to form this heterodimer, IDH1-R132H could dominant negatively inhibit IDH1-WT if a large amount of IDH1-WT molecules in the cell bind to IDH1 mutants to form heterodimers, rather than binding to other IDH1-WT molecules to form WT:WT homodimers. The fact that we did not observe dominant negative inhibition (Fig. 2b,c,d) suggests that a low proportion of IDH mutant:WT heterodimers exist in cells compared to WT:WT homodimers.

Why would few IDH WT:mutant heterodimers form in cells compared to WT:WT homodimers? Differences in binding affinity between IDH WT:WT, mutant:WT, and mutant:mutant dimers could explain this phenomenon. For instance, if IDH mutant:WT binding is weaker than mutant:mutant or WT:WT binding, formation of the WT:WT and mutant:mutant homodimers would outcompete formation of mutant:WT heterodimers. This is the case for IDH2, since IDH2 R172 mutants have a weaker IDH2-WT binding affinity than IDH2-WT itself (Fig. 1c). A difference in binding to IDH1-WT was not observed between IDH1 R132 mutants and IDH1-WT by immunoprecipitation, although we cannot rule out that a more subtle difference exists that was not detected by this method.

While IDH mutants did not exert a dominant negative effect in this system, our results show that 2HG production is a shared function of the IDH mutants. In accordance with previous reports for IDH1-R132H [15] and IDH2-R172K [6], we show that six other IDH mutants produce 2HG when overexpressed in mammalian cells (Fig. 3a,b). This is supported by the novel finding that tumors with the rare IDH1-R132L and IDH2-R172M mutations also accumulate 2HG (Fig. 3c). IDH mutations are strongly associated with a wide range of transcriptomic and genomic alterations in gliomas [18]. However, whether neomorphic IDH activity drives these changes, how it may do so, and whether these alterations contribute to cancer pathogenesis remain unclear. Also, while 2HG production is the most striking effect of the neomorphic mutants, the consumption of NADPH or α-ketoglutarate by the mutants may also have a role in cancer.

How might IDH mutations contribute to cancer pathogenesis? Mutant IDH1 expression can up-regulate hypoxia inducible factor 1α (HIF-1α), a transcription factor that has been implicated in promoting angiogenesis and malignant behavior of cancer cells [13]. Whether HIF-1α is up-regulated as a result of lowered cellular levels of α-ketoglutarate as proposed by Zhao *et al.* [13], or due to increased 2HG levels as hypothesized by Frezza *et al*. [19], is unclear. Also, a role for HIF-1α in IDH-mutated cancer pathogenesis has been questioned based on the fact that IDH-mutated gliomas are generally not “angiogenic” as would be expected for tumors with dysregulated HIF-1α [10], and because IDH-mutated leukemias were not found to have increased expression of HIF-1α target genes [5]. Recent studies have suggested that IDH mutants impair TET2, an 5-methylcytosine hydroxylase implicated in the regulation of epigenetic changes, and that this impairment dysregulates cellular differentiation [20]. Future experiments may seek to test whether altered α-ketoglutarate or 2HG concentrations can affect HIF-1a or TET2 pathway members *in vitro*, and identify the cellular pathways necessary for the IDH mutants to exert a cancer-related phenotype. Such studies may help to pinpoint therapeutic targets for treatment of IDH-mutated cancers.

## Materials and Methods

### Ethics Statement and Patient Samples

Glioma samples (Fig. 3c) were obtained from The Preston Robert Tisch Brain Tumor Center Biorepository at Duke University and their study was approved by the Duke Institutional Review Board. Written informed consent was obtained for banking and analysis of tissue samples from all patients involved in this study. No animal work was conducted in this study. Samples were analyzed previously for tumor type and IDH mutation status [2].

### Transfection

HOG cells were described previously [21], and 293T cells were obtained from ATCC (Manassas, VA). pCMV6-Entry-IDH vectors (OriGene, Rockville, MD) were used to overexpress IDH cDNAs with C-terminal FLAG-MYC epitopes. pEGFP-N1 (Clontech, Mountain View, CA) was used to express IDH1 cDNAs with N-terminal EGFP. For transfection, 5×10^6^ cells were plated in a 75 cm^2^ flask 24 h before transfection with 20 µg (unless otherwise indicated) of plasmid DNA using 50 µl Lipofectamine 2000 (Invitrogen, Carlsbad, CA). Stable HOG cell lines containing IDH1 shRNA or control were constructed by transfecting HOG cells with pSuperRetro vector (OligoEngine, Seattle, WA) containing IDH1-specific hairpin or a scrambled sequence. Clones were selected with 500 µg/ml G418 (Gibco/Invitrogen, Carlsbad, CA) for 3 weeks and expanded after single cell dilution.

### Immunoblot and immunoprecipitation

For immunoblots, anti-Myc (TA100010, OriGene) was used at 1∶1000 dilution, anti-Flag (1∶1000, TA100011, OriGene) at 1∶1000 dilution, anti-IDHC (IDH1), N-20 (sc49996, Santa Cruz Biotechnology, Santa Cruz, CA) at 1∶300 dilution, anti-IDH2, W16 (sc-55668, Santa Cruz Biotechnology) at 1∶100 dilution. Anti-GAPDH (FL-335) (sc25778, Santa Cruz Biotechnology) at 1∶10,000 dilution was used as a loading control for lysates. For immunoprecipitation, 50 µl of anti-FLAG M2 affinity resin (A2220, Sigma, St. Louis, MO) was rinsed 3×10min with 1 mL 0.2% Triton-X100 PBS. 1 ml of cells were lysed 48 h post-transfection in 0.2% Triton-X100 PBS. This lysate was added to the resin, rotated at 4°C overnight, and then centrifuged at 10,000rpm, 4°C for 1 min. The pellet was washed by resuspension in 1 ml 50 mM Tris HCl, 150 mM NaCl, pH 7.4 and re-centrifuged 6x for IDH1 and 3x for IDH2. 80 µl 2x SDS 5% β-mercaptoethanol loading buffer was added to the pellet, incubated at 100°C for 5 min, and loaded on SDS-PAGE. Intensity of bands in immunoblots were quantified using ImageJ (v1.43, available at http://rsbweb.nih.gov/ij/, developed by Wayne Rasband, National Institutes of Health, Bethesda, MD) to determine the level of knockdown of IDH1 protein by shRNA expression.

### Isocitrate dehydrogenase activity assays

Cells were harvested 48 h post-transfection and homogenized in 0.02% Triton-X100 PBS. This was sonicated 3x 20s and protein concentration was quantified. For Figs. 2b and c, reactions were performed using a final 200 µl reaction volume containing 10 µg cell lysate, 33 mM Tris-Cl pH 7.5, 2 mM MnCl2, 107 µM NADP^+^, 40 µM isocitrate (D_s_-threo-isocitrate, monopotassium salt, Sigma) at room temperature. The reaction mix without isocitrate was first prepared in a 180 µl volume, checked on the spectrophotometer to confirm that there was no activity, and then started by adding 20 µl of 400 µM isocitrate. Reaction progress was monitored by A340 nm for 1 h using a PolarSTAR Optima (BMG Labtech, Offenburg, Germany). To titrate the concentration of isocitrate (Fig. 2*d*), a more sensitive, single-channel spectrophotometer (UV-2501PC, Shimadzu, Kyoto, Japan) was used to quantify low levels of activity. These reactions were performed as described above, except 1 ml total reaction mix was used, 20 µg lysate was used, 100 µl of 10x concentrated isocitrate was added to start the reaction, and the reaction was monitored for 1 min. Reactions were performed in triplicate. A no lysate control was run along with each experiment, which confirmed that activity was specific to reaction mixes that contain lysate (not shown). NADPH production was calculated using an NADPH extinction coefficient of 6.2×10^3^ M^-1^ cm^-1^.

### 2HG analysis

Quantification of 2-HG in media/tissues was performed by LC-negative electrospray ionization-MS/MS as published previously [17] with modifications to accommodate different sample matrices. A racemic mixture of 2HG-d4 was prepared by mixing 1 mg α-ketoglutarate-d6 (Sigma/Isotec) with 1 mg NaBH_4_ (Sigma) in 0.2 mL anhydrous MeOH (Sigma) followed by 30 min incubation at 60°C.

Media above cells was collected 0, 24, and 48 h after transfection. Lysates were collected 48 hours after transfection. To 20 µL of media or lysate, 2 µL of 65 µg/mL of each 2HG-d4 enantiomer (internal standard) in water was added and the mixture dried by vacuum centrifuge (50°C, 15 min). Dry residue was treated with 50 mg/mL freshly prepared diacetyl-L-tartaric anhydride (Sigma) in dichloromethane/glacial acetic acid (4/1 by volume) and heated (75°C 30 min). After drying (50°C, 15 min) the residue was dissolved in 100 µL LC mobile phase A (see below) for analysis. For patient glioma tissue samples, 20 mg tissue, 200 µL deionized water, 1 mL chloroform, and a 4 mm ceramic bead were vigorously mixed for 45 s at speed 4 in a Fast-Prep 120 (Thermo-Savant, Waltham, MA). After centrifugation (5 min, 16,100 g), 200 µL of aqueous (upper) layer was transferred to a 1.5 mL glass vial and dried (50°C, 60 min), derivatized, and reconstituted for LC-MS/MS analysis as described above in case of media above the cells.

An Agilent 1200 series HPLC (Santa Clara, CA) was used for liquid chromatography (LC) and a Sciex/Applied Biosystems API 3200 QTrap (Carlsbad, CA) was used for triple quadrupole mass spectrometry (MS/MS). Mobile phase A: water, 3% acetonitrile, 280 µL ammonium hydroxide (∼25%), pH adjusted to 3.6 by formic acid (∼98%). Mobile phase B: methanol. Analytical column: Kinetex C_18_, 150×4.6 mm, 2.6 µm, and SafeGuard C_18_ 4×3 mm guard-column from Phenomenex (Torrance, CA). Column temperature: 45°C. Elution gradient at 1 mL/min flow rate: 0–1 min 0% B, 1–2 min 0-100% B, 2–3.5 min 100%B, 3.5–4 min 100-0% B, 4–10 min 0% B. Injection volume: 10 µL. Q1/Q3 (m/z) transitions monitored: 363/147 (2HG) and 367/151 (2HG-d4). To calibrate, 0, 0.16, 0.54, 1.8, 6, and 20 µg/ml pure (R)-2-hydroxyglutarate (Sigma) was prepared. These samples were analyzed alongside experimental samples and accuracy acceptance criteria was 85% for each but the lowest level (0.16 µg/mL, 80%). In the case of glioma tissue samples, quantification was done by using the signal resulting from the known concentration of 2HG-d4 internal standard added to the sample prior to the sample processing and analysis. A two-tailed Student's t test assuming equal variances was used to test for a significant difference in mean 2HG concentration between groups of samples.

## Supporting Information

Figure S1 **Co-expression of IDH1-R132H with IDH1-WT does not lower NADP^+^-IDH activity more than vector alone.** HOG cells were transfected with the indicated amounts of pCMV6 vectors to express IDH1-WT, IDH1-R132H, or vector alone (V), as indicated. 48 hours after transfection, cells were lysed and total cellular NADP+-IDH activity was determined at 1.3mM isocitrate. Results are from two independent measurements of lysates and are representative of two independent experiments.
(PDF)
