# Supplementary material for: 2-Hydroxyglutarate Production, but Not Dominant Negative Function, Is Conferred by Glioma-Derived NADP+-Dependent Isocitrate Dehydrogenase Mutations
Source: PLoS One. 2011 Feb 4;6(2):e16812. doi: 10.1371/journal.pone.0016812 (PMC3033901; doi:10.1371/journal.pone.0016812)
Supplement: Figure S1 — Co-expression of IDH1-R132H with IDH1-WT does not lower NADP+-IDH activity more than vector alone. HOG cells were transfected with the indicated amounts of pCMV6 vectors to express IDH1-WT, IDH1-R132H, or vector alone (V), as indicated. 48 hours after transfection, cells were lysed and total cellular NADP+-IDH activity was determined at 1.3mM isocitrate. Results are from two independent measurements of lysates and are representative of two independent experiments. (PDF) [file pone.0016812.s001.pdf]

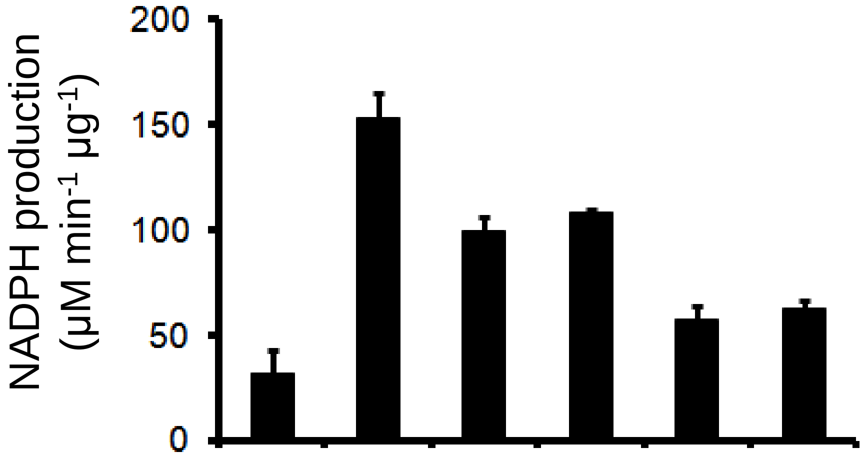

IDH1-WT	-	2 µg	2 µg	2 µg	2 µg	2 µg
V	2 µg	-	2 µg	-	6 µg	-
IDH1-R132H	-	-	-	2 µg	-	6 µg

Figure S1. Co-expression of IDH1-R132H with IDH1-WT does not lower $NADP^+$-IDH activity more than vector alone. HOG cells were transfected with the indicated amounts of pCMV6 vectors to express IDH1-WT, IDH1-R132H, or vector alone (V), as indicated. 48 hours after transfection, cells were lysed and total cellular NADP+-IDH activity was determined at 1.3mM isocitrate. Results are from two independent measurements of lysates and are representative of two independent experiments.
